# Ion-Triggered In Situ Gel Combined with Melatonin Liposomes: Breaking Through the Dual Barriers of Nasal and Brain Delivery to Treat Insomnia

**DOI:** 10.3390/pharmaceutics18060656

**Published:** 2026-05-27

**Authors:** Zhewen Dong, Xinxin Dong, He Wang, Yujie Pan, Meiqi Yang, Sihan Zhao, Wanxian Deng, Mengshan Han, Tiantian Ye, Shujun Wang

**Affiliations:** Department of Pharmaceutics, College of Pharmacy, Shenyang Pharmaceutical University, Shenyang 110016, China

**Keywords:** melatonin, liposomes, ion-triggered in situ gel, intranasal administration, nose-to-brain delivery, insomnia

## Abstract

**Background/Objectives:** Insomnia severely impairs quality of life. Oral melatonin (MEL) suffers from poor brain delivery. Intranasal administration bypasses the blood–brain barrier, but rapid mucociliary clearance shortens drug retention, and MEL poor water solubility limits its nasal dissolution. Traditional in situ gels have “gelation-first, spreading-second” defects, causing uneven distribution. Herein, we developed a two-step sequential ion-triggered in situ gel combined with MEL liposomes (MEL-Lips-Gel) to enhance solubility, achieve instant uniform coating, and prolong retention for efficient nose-to-brain delivery. **Methods:** MEL-Lips were dispersed in alginate (first component) and calcium gluconate served as the second component. After sequential spray, the two components mix and form an ion-crosslinked gel. Rheology, in vivo fluorescence imaging, in vitro release, open-field/sucrose preference tests, and H&E staining were performed. **Results:** MEL-Lips showed uniform size and good encapsulation. The sequential system achieved instant widespread spreading and rapid gelation, significantly prolonged nasal retention, enabled sustained brain delivery, and reversed insomnia-induced hyperactivity and anxiety-like behaviors more effectively than oral MEL, intranasal MEL solution, liposomes alone, or non-liposomal gel, with good nasal safety. **Conclusions:** This sequential ion-triggered liposome-in-gel strategy synergistically overcomes rapid clearance (via gel) and poor solubility (via liposomes), enhancing nose-to-brain delivery of melatonin and providing a promising platform for insomnia therapy.

## 1. Introduction

Insomnia is a prevalent sleep disorder that severely impacts the physical and mental health and quality of life of millions worldwide [1,2,3]. Melatonin (MEL), an endogenous hormone secreted by the pineal gland that primarily regulates circadian rhythms, has become a first-line clinical choice for treating insomnia [4,5,6]. However, the currently marketed oral route has significant limitations: pronounced first-pass effects lead to low bioavailability and high inter-individual variability [7,8,9]; simultaneously, the blood–brain barrier restricts its efficient delivery to the central nervous system, often requiring higher doses to achieve therapeutic concentrations in the brain, thereby increasing the risk of potential side effects [10,11,12,13].

For the treatment of insomnia, rapid onset of action is particularly critical, as patients need to fall asleep soon after drug administration. Intranasal administration offers an attractive alternative [14,15,16]. This is specifically because this route enables direct nose-to-brain transport via the olfactory and trigeminal nerves, allowing drugs to bypass the blood–brain barrier and enter the brain tissue and cerebrospinal fluid directly or rapidly, while significantly reducing systemic exposure and minimizing the next-day somnolence side effect [17,18,19]. However, traditional nasal solutions or suspensions face severe challenges from the nasal cavities’ inherent defense mechanisms: the efficient mucociliary clearance system typically removes most of the formulation within 15–20 min, greatly shortening the contact time between the drug and the absorption site, severely limiting its bioavailability and efficacy [20,21]. Moreover, MEL itself has poor water solubility, which further limits its dissolution and release in the nasal cavity, thereby compromising absorption efficiency [22].

Liposomes, as biocompatible nanocarriers, can significantly improve the water solubility of poorly soluble drugs and confer sustained-release properties [23,24,25]. Encapsulating MEL into liposomes can enhance its dissolution behavior in nasal secretions while achieving a slow drug release, thereby prolonging the duration of action. However, when liposomes are administered intranasally alone, they still encounter the problem of rapid clearance by the mucosal cilia, making it difficult to fully utilize their sustained-release advantages.

To overcome the challenge of rapid clearance, in situ Gelling delivery systems have emerged. These formulations are administered as solutions and undergo a phase transition triggered by the physiological environment of the nasal cavity (e.g., temperature, ionic strength, or pH change), forming an adhesive Gel that significantly prolongs retention time [26,27,28]. Among various Gel systems, thermosensitive Gels have been widely studied. Their Gels rely on body temperature, offering convenience of use [29,30]. However, their main drawbacks are that the Gel process may take several minutes, during which the low-viscosity precursor solution may still be partially cleared; and their Gel strength is sensitive to temperature changes, potentially being affected by ambient temperature and local airflow within the nasal cavity [31].

In contrast, ion-sensitive Gels, particularly those based on the natural polysaccharide sodium alginate (SA) crosslinked with divalent cations (e.g., Ca^2+^), exhibit unique advantages. They exhibit clear kinetic and biocompatibility advantages: sodium alginate is a linear anionic polysaccharide with abundant guluronic acid units in its molecular chains, enabling ultrafast ionic crosslinking with Ca^2+^ via the classic “egg-box” complexation, which results in rapid gelation kinetics and controllable phase transition; calcium gluconate, a weakly acidic calcium salt, gently releases Ca^2+^ and avoids local irritation. Furthermore, sodium alginate is an FDA-approved pharmaceutical excipient that is biodegradable, highly mucoadhesive, and non-immunogenic; calcium gluconate is clinically safe medicinal calcium preparation with no irritancy to nasal tissues. The combination of the two exhibits excellent biocompatibility for intranasal drug delivery [32,33,34,35]. However, the traditional application method typically involves dissolving the drug into a SA solution and administering it after pre-mixing with a calcium ion source. The fatal flaw of the pre-mixing method is that Gel formation begins before the formulation leaves the dropper or spray nozzle. This results in a highly viscous Gel precursor whose flow and spreading ability are severely diminished at the moment it contacts the nasal mucosa, making it difficult to achieve uniform and widespread distribution within the complex physiological structure of the nasal cavity [36,37]. The drug often deposits in the vestibular region, failing to effectively cover the olfactory and respiratory regions rich in absorbing cells, creating an inherent contradiction between “retention” and “distribution”.

To address this core contradiction and fully exploit the solubilizing and sustained-release advantages of liposomes, this study innovatively designed a melatonin liposome-loaded intranasal in situ gel based on a sequential ion-triggering mechanism (MEL-Lips-Gel). The essence of this gel design lies in “spreading first, solidification second”: first, the low-viscosity drug-loaded sodium alginate solution (containing MEL-Lips) achieves instant and widespread mucosal coating within the nasal cavity like a conventional solution; subsequently, the sprayed calcium ion source solution immediately triggers a rapid ion-crosslinking reaction with the already spread polymer layer, forming a stable, adhesive hydrogel layer in situ at the coating site, thereby achieving prolonged retention while locking the liposomes within the gel network for further synergistic sustained release. In this study, we first optimized and characterized the formulation and key rheological properties of this ion-triggered gel. Through visual experiments, we demonstrated its dual advantages over conventional methods in terms of initial coating uniformity and long-term retention capacity. Furthermore, we evaluated its in vitro drug release behavior and in vivo brain-targeting efficiency. Finally, we comprehensively assessed its therapeutic efficacy and local nasal safety in a mouse model of insomnia to validate its potential as an efficient nose-to-brain delivery platform.

## 2. Materials and Methods

### 2.1. Materials

Melatonin (MEL) was purchased from Shanghai Aladdin Biochemical Technology Co., Ltd. (Shanghai, China). Indocyanine green (ICG) was purchased from Shenyang Xintaigeer Pharmaceutical Technology Development Co., Ltd. (Shenyang, China). Sodium alginate (SA) was purchased from Tianjin Beichen Fangzheng Reagent Factory (Tianjing, China). Anhydrous calcium gluconate was purchased from Shanghai Macklin Biochemical Technology Co., Ltd. (Shanghai, China). Methylene blue (MB) was purchased from Shanghai Yiji Industrial Co., Ltd. (Shanghai, China). Soybean lecithin was obtained from Germany Lipoid, and cholesterol was purchased from Guangzhou Baiyunshan Pharmaceutical Co., Ltd. (Guangzhou, China). Phosphate buffered saline (PBS, pH 7.4, 0.01 M), absolute ethanol, physiological saline (0.9% NaCl), and other analytical grade chemical reagents were purchased from Beijing Solarbio Science & Technology Co., Ltd. (Beijing, China).

### 2.2. Animals

Male KM mice (aged 6–8 weeks) were supplied by Liaoning Changsheng Biotechnology Co., Ltd. (Benxi, China). The mice were housed in a specific pathogen-free (SPF) facility under a 12 h light/dark cycle and acclimatized for one week prior to experimentation. All animal experimental procedures were conducted in accordance with the Guiding Principles for the Use and Care of Experimental Animals approved by the National Science and Technology Commission of China. All animal procedures were approved by the Animal Ethics Committee of Shenyang Pharmaceutical University (approval number: SYPU-IACUC-S2024-0410-104). Food and water were available ad libitum throughout the study. Mice were anesthetized by inhalation of 5% isoflurane prior to surgical procedures.

### 2.3. Preparation and Characterization of MEL-Lips

MEL-Lips were prepared by thin-film hydration method: 45 mg of MEL, 900 mg of soybean lecithin, and 45 mg of cholesterol were accurately weighed and ultrasonic dissolved into 10 mL of Ethanol absolute, and evaporated at 40 °C in a vacuum rotator (Gongyi Instrument Co., Ltd., Shanghai, China) for 30 min to remove the Ethanol, and obtained the dried lipid deposit. Then, 10 mL of preheated aqueous solution was added into the eggplant-type flask and hydrated at 40 °C for 15 min. After hydration, the suspension was sonicated in an Ultrasonic Cell Disruptor (Ningbo Xinzhi Biotechnology Co., Ltd., Ningbo, China) with an ice bath probe for 400 W (10 min), then passed through 0.8 μm microporous membrane once, and then through 0.22 μm microporous membrane twice to obtain MEL-Lips with a concentration of 4.5 mg/mL. ICG-Lips were prepared using the same procedure as MEL-Lips, at a concentration of 1 mg/mL The prepared MEL-Lips and ICG-Lips were diluted to a specific concentration by purified water, dropped on a copper grid, and stained with 3% phosphotungstic acid. The excess liquid was absorbed by filter paper and then air-dried. The morphology was observed and photographed by transmission electron microscopy (TEM). MEL-Lips were placed at 4 °C for 7 days, and the Particle size, polydispersity index (PDI) and Zeta potential were measured using Laser Particle Analyzer (Nano-ZS90 nanosizer, Malvern Instruments, Worcestershire, UK) at 0, 3, and 7 days. Encapsulation efficiency (EE%) was determined by separating free melatonin via ultracentrifugation (100,000 rpm for 1 h at 4 °C), and the free drug content in the supernatant was measured using UV spectrophotometry.

EE (%) = Wencapsulated/Wtotal × 100

### 2.4. Formulation Screening of MEL-Lips-Gel

Solution A (MEL-Lips containing SA solution): 2% (*w*/*v*) SA solution was first prepared and placed on a magnetic stirrer, then heated to 80 °C to ensure complete dissolution. After cooling, the MEL-Lips suspension was mixed with the 2% (*w*/*v*) SA solution at a 1:1 volume ratio to obtain a final alginate concentration of 1% (*w*/*v*). The mixture was gently stirred to form a clear, homogeneous, and bubble-free solution.

Solution B (calcium gluconate solution): Aqueous solutions of calcium gluconate at concentrations of 1%, 2%, 3%, 4%, and 5% (*w*/*v*) were prepared.

Equal volumes of Solution A and Solution B were thoroughly mixed on a glass plate, and the gel formation ability as well as the gelation time were observed. After mixing, the mixture was allowed to stand for 2 min to allow complete gelation. The formed gel was then carefully separated from any residual free liquid by gentle decantation, and the unreacted components were collected and weighed on an electronic balance (Shanghai OHAUS Instruments Co., Ltd., Shanghai, China).

### 2.5. Rheological Analysis

Steady-state shear testing was performed using a rotational rheometer (Shanghai Naichi Scientific Instrument Co., Ltd., Shanghai, China): After rapid mixing of equal volumes of each formulation, it was immediately loaded onto the testing platform. The change in apparent viscosity (η) was measured within a shear rate range increasing linearly from 0.1 s^−1^ to 60 s^−1^ to evaluate the system’s shear-thinning behavior and spray suitability.

Dynamic oscillatory testing: To assess the Gel’s instantaneous formation ability and mechanical properties, frequency sweeps were performed at 25 °C and 34 °C. Frequency sweep: After the Gel formation stabilized, a scan was performed within a frequency range of 0.1–100 rad/s (strain maintained at 1%), recording the change in storage modulus (G′) and loss modulus (G″) with frequency.

### 2.6. Gel Formation and Coating Uniformity Experiment

According to the previously determined optimal formulation, the methylene blue and MEL-containing SA solution and the calcium gluconate solution were loaded into separate nasal spray bottles. They were sequentially sprayed onto a glass culture dish. After sufficient reaction, the Gel was observed and photographed to assess coating uniformity. For comparison, the same formulation was allowed to form a Gel first and then placed on a glass culture dish for observation. The coverage area was calculated and a bar chart was plotted.

### 2.7. In Vivo Nasal Retention Study in Mice

First, the fluorescence imaging capability of ICG-Lips and ICG-Lips-Gel (1 mg/mL) was evaluated using a fluorescence molecular imaging system (Beijing Digital Precision Medicine Technology Co., Ltd., Beijing, China) to validate their suitability for subsequent experiments. Six KM mice were randomly divided into two groups (n = 3): the Lips group and the Lips-Gel group. After administration, the mice were anesthetized and fixed in the supine position. Mice in the Lips-Gel group received sequential intranasal administration of 10 μL of ICG-Lips-loaded SA solution and 10 μL of calcium gluconate solution into each nostril, with an interval of approximately 5 s. The control group (Lips group) received 20 μL of ICG-Lips solution at the same concentration into each nostril. Fluorescence images of the mouse head were acquired using the fluorescence molecular imaging system at 0, 30, 60, 120, 180, and 240 min post-administration. The mean fluorescence intensity in the nasal region was semi-quantitatively analyzed using ImageJ software 10.1.2), and the fluorescence intensity-time curve was plotted.

### 2.8. In Vitro Release Study

The release test was conducted using the dialysis bag diffusion method. An equivalent of MEL-Lips-Gel containing 1% MEL was accurately weighed and placed in a pre-treated dialysis bag (molecular weight cutoff: 8000 Da). The dialysis bag was immersed in 200 mL of PBS release medium (pH 7.4, 0.01 M, consistent with the physiological pH of nasal mucus (7.0~7.6), and oscillated in a constant temperature water bath shaker (Shanghai Yiheng Scientific Instrument Co., Ltd., Shanghai, China) at 37 ± 0.5 °C and 100 rpm. The shaking speed of 100 rpm was selected to maintain gentle agitation ensuring sink conditions without causing mechanical disruption of the gel or dialysis bag. At predetermined time points (0, 1, 2, 4, 6, 8, 12 h), 2 mL of release medium was withdrawn (replaced with an equal volume of fresh pre-warmed medium). The MEL content was determined using a validated UV analytical method at a wavelength of 278 nm. MEL solution (MEL-Sol) and MEL-Lips (1 mg/mL) were used as controls. The cumulative release percentage was calculated, and release curves were plotted.

### 2.9. Brain Tissue Distribution Study

Twelve KM mice were randomly divided into 4 time-point groups (n = 3). The sequential triggered Gel labeled with ICG-Lips was administered intranasally as described above. At 30, 60, 120 and 240 min after administration, the thoracic cavity was opened to expose the heart. An injection needle was inserted into the apex of the left ventricle, the needle was fixed, the right atrial appendage was clamped, and a peristaltic pump (product of Nanjing Runze Fluid Control Equipment Co., Ltd., Nanjing, China) was started. The heart was perfused with physiological saline via the peristaltic pump until the liver turned pale and the tail became stiff. (This perfusion step ensures that the fluorescence signal detected in brain tissues reflects only drug that has entered the brain parenchyma, rather than residual blood.) After perfusion, fluorescence molecular imaging was used to obtain images of the entire brain. The whole brain was divided into four regions: olfactory bulb (OB), brainstem (BT), cerebellum (CL) and cerebrum (CB), which were photographed separately. ImageJ was used for semi-quantitative analysis of fluorescence in the olfactory bulb region.

### 2.10. Induction of Insomnia Animal Model and Treatment

Insomnia model induction: A modified pedestal sleep deprivation method was used. Model and treatment group mice were placed in a cage containing multiple small platforms (diameter 3.0 cm). The cage was filled with room-temperature water to a level approximately 1 cm below the platform surface. When mice entered rapid eye movement sleep, muscle relaxation would cause them to fall off the platform or touch the water, waking them up, thus achieving continuous sleep disturbance. The modeling period lasted for 72 consecutive hours, during which mice were removed from the platforms daily for 1 h for routine feeding, drinking, and cage cleaning. Control group mice were housed in normal cages with bedding.

Experimental grouping and administration: Mice were randomly divided into 7 groups (n = 5): (1) Control group: received daily intranasal administration of 20 μL physiological saline; (2) Insomnia model group: no treatment.; (3) a per os (*p.o.*) MEL group: based on human dose of 3 mg/60 kg, the equivalent mouse dose was 0.45 mg/kg, administered daily by oral gavage; (4) Intranasal (*i.n.*) MEL solution group: received daily intranasal instillation of MEL solution at the same dose (0.45 mg/kg) as the oral group, with an administration volume of 20 μL; (5) *i.n.* MEL-Gel group: premixed according to the optimal gel formulation, and received daily intranasal instillation of MEL-Gel containing 0.45 mg/kg melatonin, with an administration volume of 20 μL; (6) *i.n.* MEL-Lips group: Received daily intranasal instillation of MEL-Lips containing 0.45 mg/kg melatonin, with an administration volume of 20 μL; (7) *i.n.* MEL-Lips-Gel group: according to the optimal gel formulation, received daily intranasal instillation of 10 μL sodium alginate solution loaded with MEL-Lips plus 10 μL calcium gluconate solution, with a daily melatonin dose of 0.45 mg/kg.

### 2.11. Open-Field Test

The apparatus consisted of an enclosed square box (length × width × height: 40 cm × 40 cm × 30 cm). One hour after the final administration, each mouse was gently placed in the center of the enclosed box. Following a 10 min acclimatization period, the Autonomous Activity Video Analysis System V3.0 (Anhui Zhenghua Bio-Instrument Equipment Co., Ltd., Huaibei, China) was activated to record the mouse’s behavioral activities within the box for 5 min. The following parameters were collected using the V3.0 system: total distance moved and resting time, which reflect the extent of insomnia improvement in mice.

### 2.12. Sucrose Preference Test

The sucrose preference test is a well-established behavioral assay for assessing anhedonia, a core symptom of depression that is also frequently observed in chronic insomnia models. Reduced sucrose preference reflects a loss of interest in rewarding stimuli. The test was conducted on the day following the open field test. Pre-weighed bottles containing 1% sucrose solution and plain water were placed simultaneously, and the mice were allowed to drink ad libitum for 24 h. The consumption of liquid from two bottles by the mice was recorded over 24 h. The sucrose water preference rate (SPR) was calculated using the formula: SPR (%) = [Sucrose solution consumption (g)/(Sucrose solution consumption (g) + Pure water consumption (g))] × 100%.

### 2.13. Nasal Mucosa Safety Evaluation

After all behavioral tests, mice were euthanized by anesthetic overdose. The nasal tissue block containing the nasal septum and bilateral nasal turbinates was completely excised and fixed in 4% paraformaldehyde for 48 h. Standard decalcification, dehydration, and paraffin embedding were then performed. Coronal sections were cut continuously (5 μm thickness). After dewaxing and rehydration, sections were stained with Hematoxylin and Eosin (H&E). Under an optical microscope (Nikon, Tokyo, Japan), the integrity of the nasal mucosal epithelium was observed, with no significant inflammatory cell infiltration, edema, or necrosis noted.

### 2.14. Statistical Analysis

All data are expressed as mean ± standard deviation. Statistical analysis was performed using GraphPad Prism 10.1.2 software. Comparisons among multiple groups were performed using one-way analysis of variance (One-way ANOVA). A *p*-value less than 0.05 was considered statistically significant (# *p* < 0.05, ## *p* < 0.01, ### *p* < 0.001).

## 3. Results and Discussion

### 3.1. Characterization of MEL-Lips

**Figure 1:**
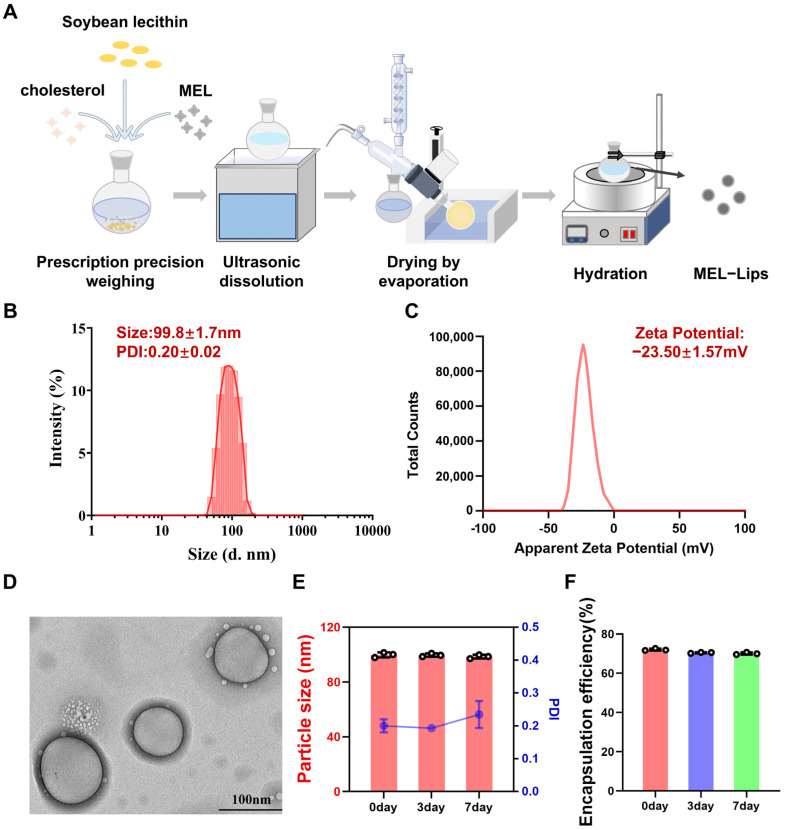
(**A**) Schematic diagram of the MEL-Lips preparation method. (**B**) Particle Size of MEL-Lips. (**C**) Zeta Potential of MEL-Lips. (**D**) TEM image of MEL-Lips. (scale bar: 100 nm) (**E**) Changes in the particle Size and polydispersity index (PDI) of MEL-Lips over 0, 3, and 7 days. (**F**) Changes in the encapsulation efficiency (EE) of MEL-Lips over 0, 3, and 7 days.

MEL-Lips were prepared by the thin-film hydration method (Figure 1A). The particle size of MEL-Lips is shown in Figure 1B. The mean diameter was 99.8 ± 1.7 nm, with a polydispersity index (PDI) of 0.20 ± 0.02, indicating a relatively uniform size distribution. As presented in Figure 1C, the zeta potential of MEL-Lips was −23.50 ± 1.57 mV. It is generally accepted that an absolute zeta potential value greater than 20 mV suggests good stability of the nanoformulation [38]; therefore, the prepared MEL-Lips exhibited satisfactory stability. TEM observation (Figure 1D) revealed that MEL-Lips were spherical or slightly irregular in shape with a clearly visible bilayer membrane, which is characteristic of typical liposome structures. Furthermore, MEL-Lips remained stable in terms of particle size and encapsulation efficiency during storage at 4 °C for up to one week (Figure 1E,F).

### 3.2. Formulation Optimization and Rheological Properties of the Gel System

**Figure 2:**
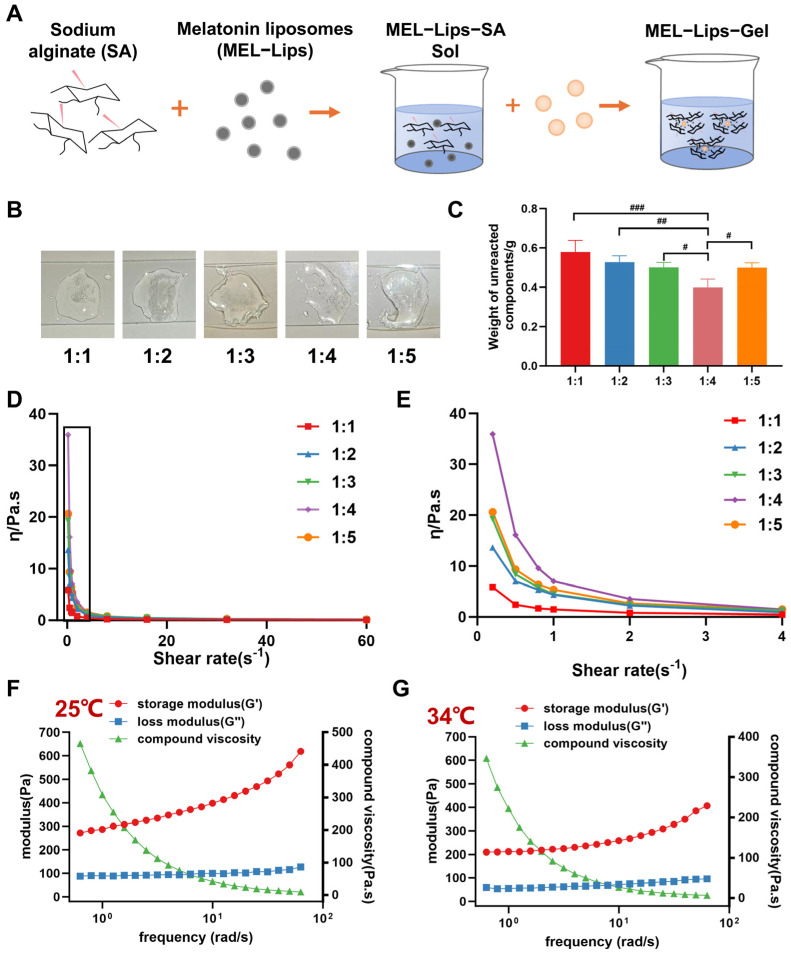
(**A**) Schematic illustration of the preparation and mechanism of the sequential ion-triggered MEL-Lips-loaded in situ nasal gel (MEL-Lips-Gel). (**B**) Visual observation of immediate Gel formation after mixing different formulations. (**C**) Quantitative analysis of the weight of unreacted components after gelation of different formulations, n = 3. (**D**) Steady-state shear rheological curve of the gels formed by different prescriptions. (**E**) Steady-state shear rheological curve the black box area in D. (**F**,**G**) Dynamic frequency sweep curves of the optimal formulation at simulated ambient (25 °C) and nasal (34 °C) temperatures. Data are mean ± SD. *p* values were calculated using one-way ANOVA, ^#^ *p* < 0.05, ^##^ *p* < 0.01, ^###^ *p* < 0.001.

The core of this study lies in constructing a MEL-Lips delivery system that undergoes rapid and controllable phase transition through the reaction between SA and calcium ions (Figure 2A). The primary task was to optimize the ratio of the core components that initiate this reaction. Through systematic formulation screening (Figure 2B,C), we found that when the *w*/*v* of the MEL-Lips containing SA solution to the calcium gluconate aqueous solution was 1:4, the gelation process was completed within 5 s, demonstrating the fastest and most complete gelation, yielding a uniform and firm gel with minimal free liquid. This ratio ensured sufficient calcium ions to drive rapid and complete crosslinking while avoiding drawbacks such as localized excessively fast Gel affecting initial fluidity or causing Gel structural inhomogeneity due to calcium ion excess.

Shear thinning is a typical non-Newtonian fluid behavior exhibited by Gel-like materials under steady-state shear [39,40]. As shown in Figure 2D,E, under simulated static nasal environment (shear rate of 0.2 s^−1^), the 1:4 ratio group exhibited the highest viscosity (approximately 36 Pa·s), indicating that this formulation maintained a high initial viscosity after gel formation. As the shear rate increased, the viscosity of all ratio groups decreased rapidly, dropping below 2 Pa·s at shear rates > 4 s^−1^. All formulations displayed pronounced shear-thinning behavior, i.e., the apparent viscosity decreased sharply with increasing shear rate. These results confirm that when the ratio of MEL-Lips-loaded sodium alginate solution to calcium gluconate solution is 1:4, the formulation is well suited for the high-shear process of nasal spraying, ensuring smooth ejection and uniform spreading within the nasal cavity.

The results of dynamic oscillatory frequency sweeps are crucial. As shown in Figure 2F,G, at room temperature (25 °C) and under simulated nasal environment temperature (34 °C), the storage modulus (G′) was significantly higher than the loss modulus (G″) at the initial frequency (0.6 rad/s), and this G′ > G″ relationship was maintained across the entire frequency range (0.6–62.8 rad/s). At 25 °C, G′ increased from approximately 270 Pa to approximately 620 Pa; at 34 °C, G′ increased from approximately 210 Pa to approximately 410 Pa, whereas G″ remained relatively stable at around 100 Pa. These quantitative observations clearly indicate that an elastic-dominated three-dimensional gel network was rapidly established immediately upon contact of the two solutions. The gel strength on the order of several hundred Pa provides the necessary mechanical strength to resist the shear forces induced by nasal mucociliary movement and mucus turnover [41]. Taken together, the above results demonstrate that the ion-triggered gel used in this study achieves an instantaneous “liquid-to-solid” transition, which is key to ensuring that the solution layer is spread and then solidified.

### 3.3. In Vivo Nasal Retention and Distribution

**Figure 3:**
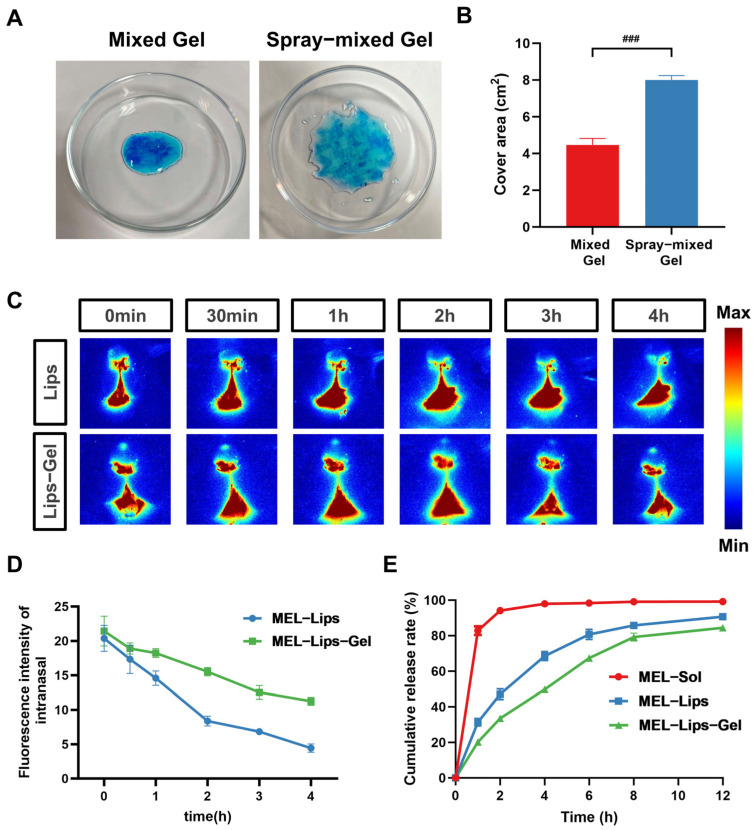
(**A**) Comparison of the coating morphology of methylene blue-labeled mixed gel and spray-mixed gel on a simulated surface. (**B**) Quantitative analysis of the coverage area of gels formed by the two methods, n = 3. (**C**) Comparison of the nasal retention of ICG-Lips and ICG Lips-Gel in mice. (**D**) Quantitative fluorescence signal decay curve in the nasal region based on fluorescence intensity analysis, n = 3. (**E**) In vitro cumulative release profiles of MEL from the MEL-Lips, MEL-Lips-Gel and a MEL-Sol, n = 3. Data are mean ± SD. *p* values were calculated using one-way ANOVA, ^###^ *p* < 0.001.

First, regarding the instant and uniform coating of the intranasal Gel, we used methylene blue to simulate the drug, directly comparing the deposition morphology formed on a simulated nasal mucosal surface by “traditional mixed Gel” and “spray-mixed Gel” (Figure 3A,B). In the pre-mixed group, gelation had already begun before spraying, resulting in irregular, small clumps with limited coverage area, indicating that large areas of the nasal mucosa remained uncovered by the drug. In contrast, in the sequential trigger group, the low-viscosity drug-loaded alginate solution was first sprayed into the nasal cavity, where its low viscosity allowed rapid flow across the nasal mucosal surface, achieving extensive coverage. After an interval of approximately 5 s, the calcium gluconate solution was then sprayed in, crosslinking with the already spread polymer layer to form a gel in situ. This resulted in a significantly larger film with uniform color and smooth edges. This morphological difference confirms the success of the “distribution first, solidification second” strategy, theoretically maximizing the contact area between the drug and the nasal absorption epithelium (especially the olfactory region), creating optimal conditions for efficient intranasal absorption.

Second, for the evaluation of in vivo nasal retention, the near-infrared dye ICG was used as a tracer [42] to prepare ICG-Lips. The prepared ICG-Lips had a particle size of 100.2 ± 1.8 nm with a PDI of 0.27 ± 0.01, which was similar to that of MEL-Lips (Supplementary Figure S1A). The morphology of ICG-Lips was that of spherical unilamellar vesicles with intact structure and a clear bilayer membrane (Supplementary Figure S1B). To verify the detectability of the fluorescence signal in vivo, ICG-Lips and an ICG solution (ICG-S) at the same concentration were imaged in vitro using a fluorescence imaging system. The results (Supplementary Figure S1C,D) showed that both formulations exhibited clear fluorescence signals, and the fluorescence intensity of ICG-Lips was comparable to that of ICG-S, indicating that the liposome encapsulation process did not cause fluorescence quenching or significant signal attenuation of ICG. Therefore, ICG-Lips were selected as the model drug for the nasal retention study.

Although intranasal administration of melatonin significantly improved bioavailability compared with oral administration, it still faces the drawback of being rapidly cleared by mucociliary clearance [43]. As shown in Figure 3C,D, after administration of the ICG-Lips-loaded sequential ion-triggered gel, the fluorescence signal intensity in the nasal region was high and decayed slowly, with approximately 52% of the initial fluorescence intensity retained after 4 h. In contrast, the fluorescence signal of the ICG-Lips group weakened rapidly within 4 h after administration, retaining only about 20% of the initial intensity. This quantitatively demonstrates that the in situ formed gel layer strongly adheres to the nasal mucosa, effectively resisting the rapid mucociliary clearance mechanism of the nasal cavity, thereby achieving the intended goal of long-term drug retention in the nose.

### 3.4. In Vitro Release Study

To evaluate whether this MEL-Lips-Gel possesses a sustained-release effect, an in vitro release study was performed. The results (Figure 3E) showed that MEL-Sol exhibited a rapid burst release, with 82.9% of the drug released within 1 h and complete release at 12 h, indicating no significant sustained-release effect. MEL-Lips displayed a mild sustained-release behavior, with a release of 31.3% at 1 h, which was significantly lower than that of the free drug solution. Thereafter, the drug was released steadily and reached equilibrium at 12 h with a cumulative release of 92.2%, reflecting the diffusion-controlled release effect of the liposomal bilayer.

MEL-Lips-Gel demonstrated the most favorable dual sustained-release characteristics, with a further reduced initial burst release of only 18.7% at 1 h, significantly lower than that of the liposome group. The entire release profile was smooth, with a continuous and stable drug release, achieving a cumulative release of 85.3% at 12 h, and the release rate was significantly slower than those of the previous two groups. The melatonin-loaded microspheres prepared by Laura et al. significantly improved the long-term retention of melatonin on the nasal mucosa [44] but lacked sustained-release properties. In contrast, our results indicate that liposomes can achieve a primary sustained release of melatonin, while the introduction of the gel further entraps the liposomes and reduces the initial burst release. Through the dual action of liposomal membrane-controlled release and gel matrix-controlled release, a long-acting and stable melatonin release is achieved, which better meets the requirements for long-acting intranasal drug delivery.

### 3.5. Brain Tissue Distribution Study

**Figure 4:**
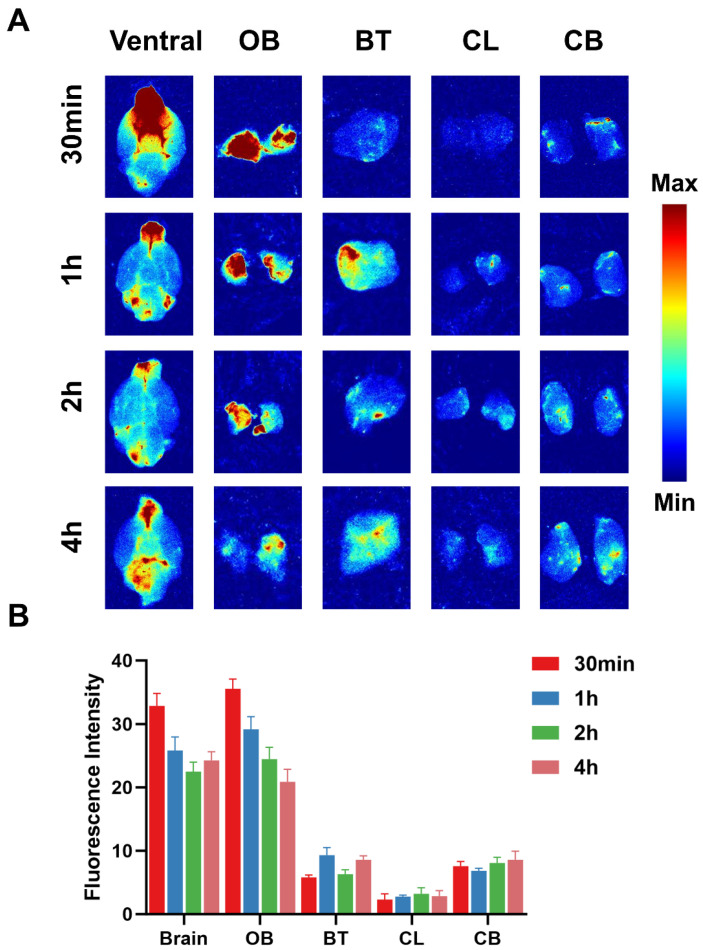
(**A**) Ex vivo fluorescence images of whole brains and dissected brain regions (olfactory bulb (OB), brainstem (BT), cerebellum (CL), cerebrum (CB)) harvested at different time points after intranasal administration of ICG-Lips-Gel, n = 3. (**B**) Semi-quantitative analysis of fluorescence intensity in various brain regions at different time points, n = 3.

Since MEL is a hormone mainly secreted by the pineal gland, efficient and sustained brain-targeted delivery efficiency is crucial [45]. Therefore, we used ICG-Lips-Gel as a model drug. Semi-quantitative fluorescence analysis of different brain regions at various time points after administration showed that in the ICG-Lips-Gel group, significant fluorescence accumulation was detected in the olfactory bulb (OB), brainstem (BT), cerebellum (CL) and cerebrum (CB) as early as 30 min post-administration, with the highest fluorescence intensity observed in the OB. More importantly, at 2 h and even 4 h after administration, calculation of the relative fluorescence percentage in each brain region further confirmed that this Lips-Gel system possesses excellent brain-targeting accumulation efficiency (Figure 4A,B). Previous studies have shown that the uptake of melatonin in the cerebrospinal fluid after intranasal administration of melatonin solution is not increased compared with intravenous injection [46], possibly because small lipophilic drugs mainly enter the brain via the systemic circulation [47]. The trigeminal and olfactory nerve pathways have recently received considerable attention as transport routes in the field of nose-to-brain delivery. Numerous studies have demonstrated that nanodrug delivery systems can efficiently transport drugs to the brain via the trigeminal and olfactory nerve pathways [48,49,50]. The OB targeting results obtained with the liposome-gel delivery system in the present study suggest that this system may achieve efficient brain-targeting efficiency through the trigeminal and olfactory nerve pathways. This reveals two major advantages of this system: (1) Rapid onset: Widespread initial distribution ensures the drug can rapidly initiate the brain entry process via the rich nasal nerves and vascular pathways; (2) Sustained distribution: The sequential ion-triggered Gel provides sustained drug supply, supporting prolonged residence of the drug in the brain.

### 3.6. Pharmacodynamic Evaluation and Safety Assessment in the Insomnia Model

**Figure 5:**
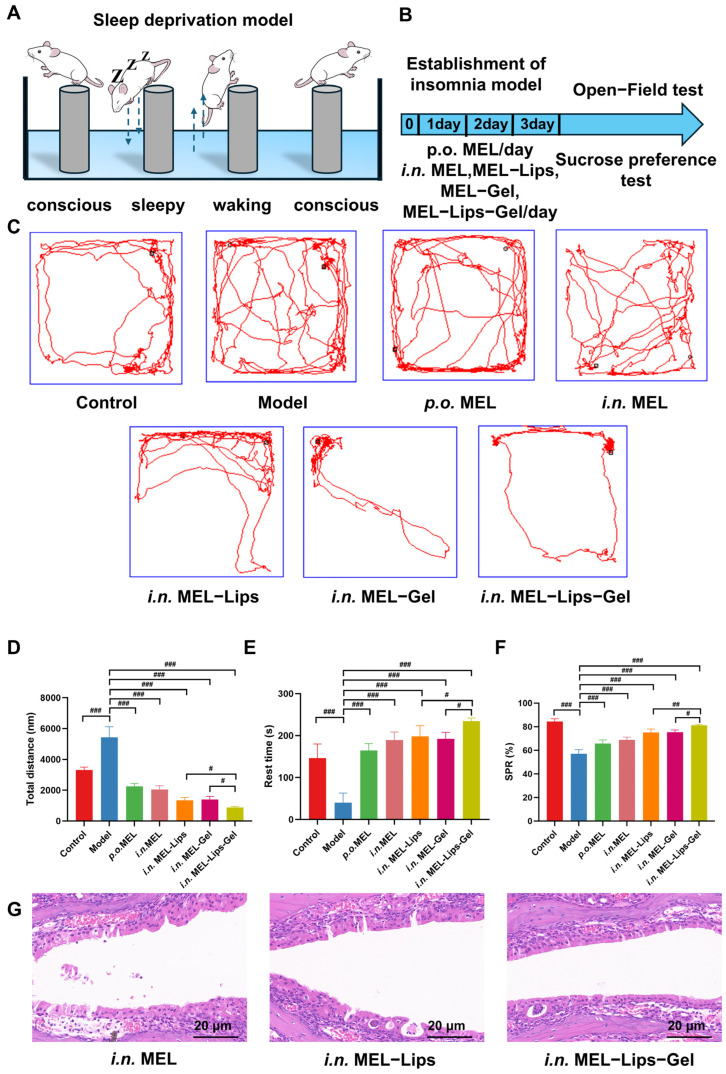
(**A**) Schematic diagram of the mouse insomnia model induced by the sleep deprivation method. (**B**) Schematic of experimental grouping and treatment protocol. (**C**) Representative movement trajectories of mice from each group in the Open-field test, n = 5. (**D**) Total distance of mice in the open field, n = 5. (**E**) Rest time of mice in the open field, n = 5. (**F**) Sucrose water preference rate (SPR) of mice in each group. (**G**) Representative images of nasal mucosal histopathological sections (H&E staining) (scale bar: 20 μm). Data are mean ± SD. *p* values were calculated using one-way ANOVA, ^#^ *p* < 0.05, ^##^ *p* < 0.01, ^###^ *p* < 0.001.

A sleep deprivation-induced insomnia mouse model was established using the horizontal platform method [51,52] (Figure 5A). The therapeutic effects of the MEL-Lips-Gel system were compared with oral MEL and intranasal MEL solution (Figure 5B). After modeling and administration, the open-field test was used to quantify the locomotor activity and anxiety-like behavior of the insomniac mice [53,54]. As shown in Figure 5C,D, the insomnia model group exhibited a typical “hyperactive” state, with a significantly increased total movement distance compared to the normal control group, and the movement trajectory maps showed chaotic activity. This phenomenon is attributed to an imbalance in central excitability caused by melatonin deficiency in the brain, further confirming the successful establishment of the insomnia model.

Both the *p.o.* MEL group and the *i.n.* MEL solution group showed certain improvements, with total movement distances reduced by 58.2% and 61.8% compared to the model group, respectively (*p* < 0.001). However, the efficacy of oral administration was limited by first-pass metabolism, while the nasal solution was affected by rapid mucociliary clearance. The *i.n.* MEL-Lips group and the *i.n.* MEL-Gel group exhibited comparable therapeutic effects, with total movement distances reduced by 74.6% and 73.8% (*p* < 0.001), respectively, and no significant difference between the two groups. Notably, the *i.n.* MEL-Lips-Gel group showed the most prominent therapeutic effect, which was significantly superior to both the *i.n.* MEL-Lips and *i.n.* MEL-Gel groups (*p* < 0.05). Further anxiety-related indicators (reduced rest time) also showed that the *i.n.* MEL-Lips-Gel group achieved the best reversal effect (Figure 5E), consistent with the previous trends. The sucrose preference test, which was further used to assess anhedonia-like behavior [55,56], yielded consistent results (Figure 5F). Collectively, these behavioral data strongly demonstrate that the MEL-Lips-Gel, through its advantages of prolonged retention, uniform distribution, and sustained release, enables more efficient and stable central drug delivery, thereby enhancing the therapeutic efficacy of melatonin. All treatment groups in this study received the same dose (0.45 mg/kg), converted from the human dose of 3 mg/60 kg based on body surface area. Oral melatonin has a bioavailability of only approximately 10–15% due to the first-pass effect, whereas intranasal administration bypasses hepatic metabolism. The pharmacodynamic results of this study showed that the therapeutic effect in the MEL-Lips-Gel group was significantly higher than that in the control group, and together with the brain fluorescence data presented above, these findings demonstrate that this formulation significantly improves the bioavailability of melatonin.

The core pathological mechanism of insomnia is closely associated with melatonin receptors, gamma-aminobutyric acid (GABA), serotonin, and 5-hydroxytryptamine (5-HT) overexpression [57], all of which collectively led to the typical behavioral phenotype of hyperactivity and anxiety in mice. Therefore, the behavioral changes observed in this study—reduced locomotor activity, prolonged resting time, and restored sucrose preference—indirectly reflect the restorative effect of MEL-Lips-Gel on neuro-related biochemical indicators.

All therapeutic improvements are based on good biosafety. Given the clinical need for short-term on-demand administration for insomnia, this study evaluated the short-term safety of the nasal mucosa using H&E staining. Histopathological (H&E) examination of the nasal mucosa showed that after administration of MEL-Lips-Gel, the nasal epithelial structure of mice remained intact, with no obvious signs of inflammatory cell infiltration, edema, or necrosis (Figure 5G). These findings confirm the favorable short-term biocompatibility and local tolerance of the sodium alginate-calcium ion crosslinking system. However, clinical translation will require longer-term toxicity data, which should be systematically investigated in subsequent large-animal preclinical studies.

## 4. Conclusions

In this study, we successfully developed a sequential ion-triggered in situ gel embedding melatonin liposomes (MEL-Lips-Gel) for enhanced nose-to-brain delivery and insomnia therapy. The liposomes improved melatonin solubility and provided a primary sustained-release effect, while the ion-triggered gel enabled instant uniform coating and significantly prolonged nasal retention. The optimized gel exhibited rapid gelation, favorable shear-thinning behavior, and sustained drug release through dual membrane-matrix control. Following intranasal administration, MEL-Lips-Gel achieved efficient and sustained delivery of melatonin to multiple brain regions, including the olfactory bulb, brainstem, cerebrum, and cerebellum, for up to 4 h. In a mouse model of insomnia, MEL-Lips-Gel significantly outperformed oral melatonin, intranasal melatonin solution, liposomes alone, and gel alone in reversing hyperactivity, anxiety-like behaviors and pleasure loss caused by insomnia, with good nasal mucosal safety confirmed by histology. Collectively, this liposome-in-gel platform integrates instant widespread coating, prolonged retention, sustained release, and enhanced solubility, offering a promising and safe strategy for intranasal treatment of insomnia and other central nervous system disorders.

The two components sequential ion-triggered system developed in this study adopts an independent storage design for the two components, which completely prevents premature ionic crosslinking and gelation during storage, thereby ensuring storage and clinical handling stability from a formulation structural perspective. This study validated the short-term refrigerated stability of MEL-Lips. Both sodium alginate and calcium gluconate are conventional pharmaceutical excipients with stable physicochemical properties, and can remain in a homogeneous state under refrigerated conditions for extended periods, meeting the storage, transport, and clinical turnaround requirements of the formulation. In actual administration, this sequential nasal spray is simple to operate, requiring only a short interval between sprays to complete the dosing procedure. When combined with a dual-chamber integrated nasal spray bottle, the operation can be further simplified and potential patient compliance issues eliminated. Furthermore, all excipients used in this liposome–gel combination system are safe pharmaceutical excipients listed in pharmacopoeias both domestically and internationally. The preparation process involves only well-established pharmaceutical technologies such as thin-film dispersion, solution preparation, and sterile filtration, allowing standardized scale-up of the manufacturing process. The formulation exhibits high regulatory compliance and low industrialization barriers. The associated manufacturing and regulatory challenges can be addressed by conventional pharmaceutical industrial approaches, indicating favorable prospects for clinical translation and manufacturing application.

## Data Availability

Data will be made available on request.

## Supplementary Materials

The following supporting information can be downloaded at: https://www.mdpi.com/article/10.3390/pharmaceutics18060656/s1. Supplementary Figure S1. (A) Particle Size of ICG-Lips. (B) TEM image of ICG-Lips. (scale bar: 100 nm) (C) The fluorescence imaging capabilities of ICG-Lips and ICG solution (ICG-S). (D) The fluorescence intensity comparison between ICG-Lips and ICG-S, n = 3.
