# Supplementary material for: Ion-Triggered In Situ Gel Combined with Melatonin Liposomes: Breaking Through the Dual Barriers of Nasal and Brain Delivery to Treat Insomnia
Source: Pharmaceutics. 2026 May 27;18(6):656. doi: 10.3390/pharmaceutics18060656 (PMC13306182; doi:10.3390/pharmaceutics18060656)
Supplement: Supplementary file 1 [file pharmaceutics-18-00656-s001.zip › pharmaceutics-4323486-supplementary.pdf]

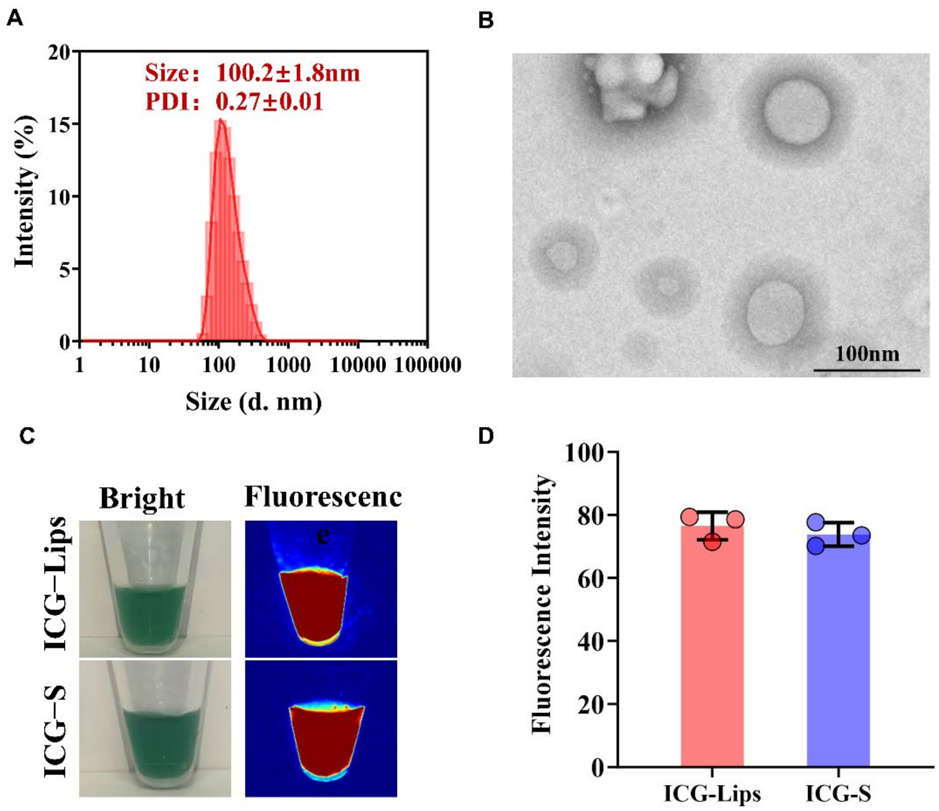

Supplementary Figure S1 (A) Particle Size of ICG-Lips. (B)TEM image of ICG-Lips. (scale bar: 100 nm) (C) The fluorescence imaging capabilities of ICG-Lips and ICG solution (ICG-S). (D) The fluorescence intensity comparison between ICG-Lips and ICG-S, n=3.
